# In vivo PIWI slicing in mouse testes deviates from rules established in vitro

**DOI:** 10.1261/rna.079349.122

**Published:** 2023-03

**Authors:** Mark Dowling, David Homolka, Nicole Raad, Pascal Gos, Radha Raman Pandey, Ramesh S. Pillai

**Affiliations:** Department of Molecular Biology, University of Geneva, CH-1211 Geneva 4, Switzerland

**Keywords:** MIWI, MILI, pachytene piRNA, slicer, PIWI, cleavage-site mismatch

## Abstract

Argonautes are small RNA-binding proteins, with some having small RNA-guided endonuclease (slicer) activity that cleaves target nucleic acids. One cardinal rule that is structurally defined is the inability of slicers to cleave target RNAs when nucleotide mismatches exist between the paired small RNA and the target at the cleavage site. Animal-specific PIWI clade Argonautes associate with PIWI-interacting RNAs (piRNAs) to silence transposable elements in the gonads, and this is essential for fertility. We previously demonstrated that purified endogenous mouse MIWI fails to cleave mismatched targets in vitro. Surprisingly, here we find using knock-in mouse models that target sites with cleavage-site mismatches at the 10th and 11th piRNA nucleotides are precisely sliced in vivo. This is identical to the slicing outcome in knock-in mice where targets are base-paired perfectly with the piRNA. Additionally, we find that pachytene piRNA-guided slicing in both these situations failed to initiate phased piRNA production from the specific target mRNA we studied. Instead, the two slicer cleavage fragments were retained in PIWI proteins as pre-piRNA and 17–19 nt by-product fragments. Our results indicate that PIWI slicing rules established in vitro are not respected in vivo, and that all targets of PIWI slicing are not substrates for piRNA biogenesis.

## INTRODUCTION

Argonautes are small RNA-binding proteins that function in most organisms and in all cells of multicellular organisms (Carmell et al. 2002). PIWI proteins belong to an animal-specific clade of Argonautes that are almost exclusively expressed in the gonads, and they associate with the small RNAs called PIWI-interacting RNAs (piRNAs) (Aravin et al. 2006; Girard et al. 2006; Grivna et al. 2006; Vagin et al. 2006). One major target of piRNAs is the collection of transposable elements present within the genome, and they are repressed by posttranscriptional or cotranscriptional mechanisms mediated by the PIWI proteins. Consistently, animals (flies, fish, mice, and hamsters) that lack either PIWI proteins or piRNA pathway factors derepress transposable elements, leading to arrested gametogenesis and infertility (Ghildiyal and Zamore 2009; Luteijn and Ketting 2013).

Three mouse PIWI proteins are expressed in the male germline, with MIWI (PIWIL1) (Deng and Lin 2002; Reuter et al. 2011) and MILI (PIWIL2) (Kuramochi-Miyagawa et al. 2004; Aravin et al. 2006) being cytosolic, while MIWI2 (PIWIL4) is nuclear (Carmell et al. 2007; Aravin et al. 2008; Kojima-Kita et al. 2016). MIWI2 and MILI are expressed in the embryonic male germline where they associate with piRNAs that almost exclusively target transposable elements (Aravin et al. 2007, 2008; De Fazio et al. 2011; Xiol et al. 2012). In the postnatal meiotic pachytene spermatocytes and postmeiotic round spermatids, MILI and MIWI associate with a distinct set of small RNAs called the pachytene piRNAs (Aravin et al. 2006; Girard et al. 2006). They are composed of highly unique sequences that are largely depleted of transposable element sequences. Although pachytene piRNAs are very abundant, most lack any obvious perfectly complementary targets in the transcriptome. Searching for genuine targets of pachytene piRNAs with a view of explaining their functions has been a major preoccupation in the field.

Mouse MIWI is exclusively bound by pachytene piRNAs and its loss leads to male infertility (Deng and Lin 2002; Reuter et al. 2011). Pachytene piRNAs originate from ∼100 large genomic loci called piRNA clusters (Li et al. 2013). Pointing to the relevance of these small RNAs, loss of thousands of piRNAs from two such clusters leads to male infertility (Wu et al. 2020; Choi et al. 2021). However, not all clusters seem to be important for fertility when individually deleted (Homolka et al. 2015; Wu et al. 2020). Pachytene piRNAs are also shown to regulate germline gene expression by identifying mRNAs via partial complementarity pairing (via the 5′ seed sequence; piRNA nucleotides 2–8) to recruit the deadenylation machinery for promoting RNA decay (Gou et al. 2014), a mechanism similar to that used by microRNAs (Filipowicz et al. 2008).

One core function of some of the Argonaute proteins is their small RNA-guided endoribonuclease (slicer) activity (Liu et al. 2004; Meister et al. 2004; Meister 2013). This is true for MIWI (Reuter et al. 2011) and MILI (De Fazio et al. 2011), with loss of their slicer activities leading to male infertility. Pachytene piRNA-guided slicer activity is shown to cleave transposon transcripts (Reuter et al. 2011; Goh et al. 2015) and mRNAs (Goh et al. 2015; Wu et al. 2020). However, the number of such slicer targets—as defined by existing targeting rules—is very limited. PIWI slicing is also linked to piRNA biogenesis, where one of the target cleavage fragments matures as the so-called secondary piRNA (Brennecke et al. 2007; Gunawardane et al. 2007). Slicing can also initiate phased piRNA biogenesis, where additional piRNAs are generated downstream from the secondary piRNA by the action of the endonuclease Zucchini/MitoPLD (Izumi et al. 2020). Slicing-triggered phasing is demonstrated in the fly ovarian germline (Han et al. 2015; Homolka et al. 2015; Mohn et al. 2015; Wang et al. 2015), and by MILI in the mouse embryonic male germline (Yang et al. 2016; Wenda et al. 2017). A similar biogenesis role for pachytene piRNA-guided slicing of cluster precursor transcripts is proposed (Gainetdinov et al. 2018; Wu et al. 2020). Taken together, pachytene piRNA-guided PIWI slicing has a function in regulating target transcript levels and in initiating piRNA biogenesis.

In this study, we sought to experimentally determine the in vivo consequence of pachytene piRNA-guided slicer activity on an endogenous mRNA. Using MIWI slicing rules established in vitro (Reuter et al. 2011), we designed two knock-in mouse lines (with a single binding site or ten binding sites) where the piRNA is perfectly complementary to the target site. This resulted in target slicing, as expected (Reuter et al. 2011). We also made two negative control knock-in mouse lines where the binding site for the piRNA on the target is mutated to result in a 2-nt mismatch at the cleavage site (nucleotides 10 and 11 of the piRNA) to prevent slicing, as previously shown in vitro (Reuter et al. 2011). Surprisingly, these negative control targets are also sliced in vivo, upending one of the sacrosanct rules of Argonaute/PIWI slicing. Our study should prompt a revision of the current piRNA-target engagement rules, which are largely based on in vitro slicing assays.

## RESULTS AND DISCUSSION

### Knock-in mice expressing mRNAs with pachytene piRNA binding sites

**FIGURE 1.**
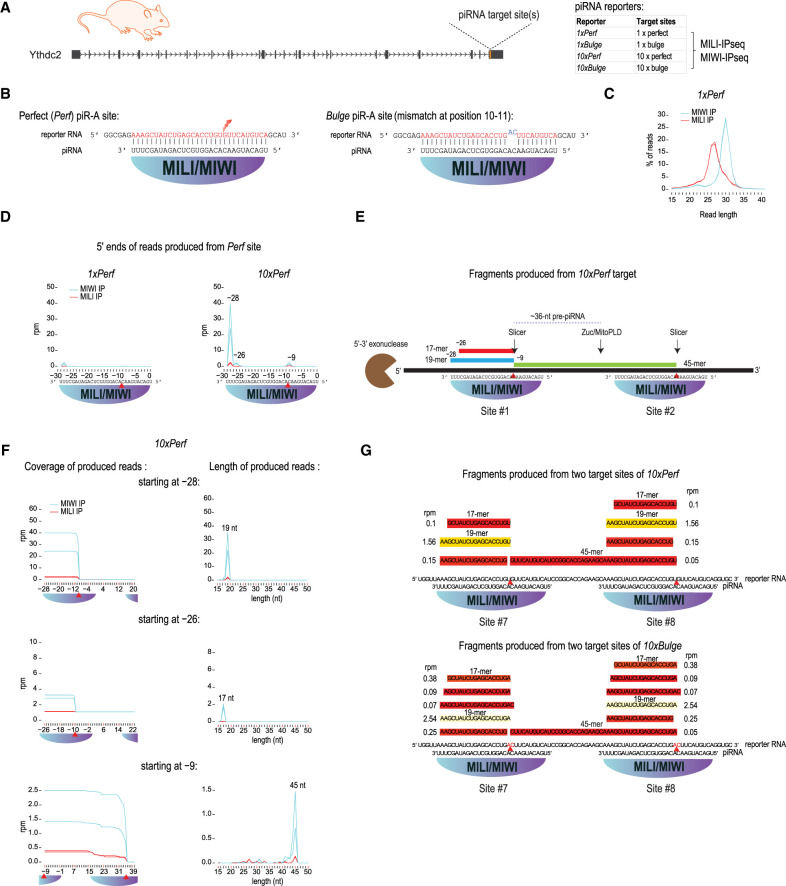
Products of target RNA slicing remain bound to PIWI complexes in mouse testes. (*A*) Perfect or bulge piR-A target sites were inserted into the last exon of *Ythdc2*. Single or 10 tandem sites with spacers were inserted. MILI and MIWI loaded sequences were identified by sequencing of immunoprecipitated RNAs. (*B*) Perfect (*Perf*) piR-A sites were designed to be fully complementary to endogenous piR-A piRNA. The bulge piR-A sites were designed to contain noncomplementary bases to piR-A nucleotides at positions 10 and 11. (*C*) Length profiles of immunoprecipitated small RNAs from *1xPerf* mouse. (*D*) The position (=distance from 5′ nt of targeting piR-A) and abundance of 5′ ends of reads produced from reporter target sites. In the case of *10xPerf*, the metaplot summarizes the read counts from all ten sites. Red triangle refers to expected position of MILI/MIWI cleavage guided by piR-A. (*E*) Schematic overview of RNA fragments identified in MILI/MIWI immunoprecipitations from *10xPerf* mice. (*F*) Coverage of reads is shown separately for RNAs with their 5′ end starting at a specific distance from the first piR-A target nucleotide (=0 distance). These RNAs are of specific lengths, with the 3′ ends always created by MILI/MIWI slicing at a subsequent site. Metaplots summarize the read counts from 10 sites. (*G*) The RNAs produced from sites 7 and 8 in *10xPerf* and *10xBulge* are shown together with their abundance. Reads from MILI and MIWI immunoprecipitations are combined. Only reads with abundance ≥0.05 rpm are shown.

To study the in vivo consequence of pachytene piRNA-mediated slicing, we prepared knock-in mice where we placed binding sites for a single abundant pachytene piRNA in the 3′UTR of the *Ythdc2* mRNA (Fig. 1A). The germline-specific RNA helicase *Ythdc2* is expressed in meiotic germ cells, including pachytene spermatocytes (Wojtas et al. 2017; Jain et al. 2018). The piRNA we chose (piR-A) starts to be expressed at postnatal day 14 in mouse testes where pachytene spermatocytes are the dominant germ cell type (Girard et al. 2006; Reuter et al. 2011). It continues to increase in abundance in postmeiotic round spermatids in P20 mice and is detected in adult mouse testes. Analysis of PIWI-bound small RNAs from P14, P20 and adult mouse testes shows that piR-A is present in both MIWI and MILI complexes (Reuter et al. 2011).

We placed a single perfectly (*1xPerf*) complementary binding site for piR-A in the 3′UTR of *Ythdc2* (Fig. 1B). To increase the chance of interaction between the target and the piRNA/PIWI complexes, we also prepared mice with ten perfectly (*10xPerf*) complementary binding sites that are separated with spacer sequences between them. In vitro slicer assays with immunopurified MIWI complexes isolated from mouse testes previously showed that a target RNA that is perfectly complementary to piR-A is cleaved (Reuter et al. 2011). Since MILI is also a slicer (De Fazio et al. 2011), the *Ythdc2* mRNA with *1xPerf* and *10xPerf* target sites are expected to be sliced by MILI too. As a noncleaving control, we prepared knock-in mice with a single (*1xBulge*) or ten (*10xBulge*) mutated binding sites (Fig. 1B). The mutations result in mismatches with the 10th and 11th nucleotide of the base-paired piR-A, creating the “bulge” and preventing slicing as previously demonstrated in vitro (Reuter et al. 2011).

Knockout of *Ythdc2* causes early spermatogenic arrest in P8 mouse testes, at a time when germ cells enter meiosis (Bailey et al. 2017; Wojtas et al. 2017; Jain et al. 2018), while conditional knockout in meiotic pachytene spermatocytes also results in arrested spermatogenesis (Liu et al. 2021). Slicing of an RNA target via small RNA-guided Argonaute leads to degradation of the RNA, reducing gene expression (Meister 2013). Slicer targets of pachytene piRNAs have lower transcript levels (Goh et al. 2015; Wu et al. 2020), as the RNA is either degraded or consumed during piRNA biogenesis (Han et al. 2015; Homolka et al. 2015; Mohn et al. 2015). However, we did not observe any impact on fertility for any of the four homozygous knock-in lines with piRNA binding sites in *Ythdc2* mRNA. Although we occasionally noted reduced litter size with the *10xBulge* line which also displayed reduced expression of postmeiotic genes (Supplemental Fig. 1C), all lines were maintained in the laboratory by crossing the homozygous lines. Also, there wasn't any dramatic change in target *Ythdc2* mRNA levels (Supplemental Fig. 1B). We conclude that presence of multiple binding sites for an abundant pachytene piRNA in the 3′UTR of *Ythdc2* did not result in gross fertility defects.

### Sliced mRNA target fragments retained within PIWI complexes

To examine whether slicing of the 3′UTR of the *Ythdc2* mRNA results in generation of new piRNAs, we prepared total testicular small RNA libraries (20–40 nt). We used P14 homozygous knock-in *1xPerf* and *1xBulge* mice but did not detect any target-derived small RNAs (data not shown). Since the level of piR-A is higher in later stages of spermatogenesis we next examined adult mouse testes (Girard et al. 2006; Reuter et al. 2011). To potentially enrich for any low levels of generated small RNAs, we immunoprecipitated MILI and MIWI complexes from knock-in mouse lines and wildtype controls. Deep sequencing of the associated RNAs revealed the expected peak read length of ∼26 nt for MILI piRNAs and ∼30 nt for MIWI piRNAs (Fig. 1C).

To detect target-derived RNAs, we mapped the reads to the *Ythdc2* 3′UTR target sequences expressed in the *1xPerf* and the *10xPerf* knock-in mice (Fig. 1D). MIWI/MILI slicing of the target sequence should result in a cleavage after the 10th nucleotide (at position −9) of the targeting piRNA (Fig. 1E; Reuter et al. 2011). This is expected to generate a pre-piRNA fragment with a 5′ monophosphate that matures into a new piRNA, and a by-product fragment with a 3′ hydroxyl group at the site of cleavage that is normally degraded. Mapping of the 5′ ends identify reads that start at position −9, consistent with their origin from slicer cleavage, and others with their 5′ ends at position −26 and −28 nt downstream from the 5′ nucleotide of the targeting piRNA (Fig. 1D). Since very low levels of target-derived sequences are detected in the *1xPerf* mice, we focused our analyses on data obtained from the *10xPerf* mice.

Reads with their 5′ ends at position −9 are not in the piRNA size-range (26–30 nt), indicating that these are not mature piRNAs. Instead, they have a fixed size of 45 nt (Fig. 1E,F) and their 5′ and 3′ ends precisely map to the slicer cleavage sites on two consecutive piRNA targeting sites (Fig. 1G). This shows that while PIWI slicer activity creates the 5′ end of the pre-piRNA fragment, the downstream cleavage at ∼36 nt that is normally expected to be generated by Zuccini/MitoPLD during phased piRNA biogenesis in mice (Yang et al. 2016; Wenda et al. 2017; Gainetdinov et al. 2018; Izumi et al. 2020), did not take place. A defining feature of abundant piRNAs is a uridine (U1) at the 5′ end, with non-U1 piRNAs being rare (Genzor et al. 2021). The pre-piRNA generated from the *10xPerf* target RNA has a guanosine (G1) (Fig. 1B,G), which may be suboptimally loaded into a PIWI protein to enable efficient piRNA biogenesis (Kawaoka et al. 2011). Alternatively, we may conclude that not all MIWI/MILI slicer cleavages can lead to piRNA biogenesis.

The reads with their 5′ ends at positions −26 and −28 have defined lengths of 17 and 19 nt, respectively (Fig. 1F), with their 3′ end at the slicer cleavage site (Fig. 1E,G). This identifies these fragments as the by-product fragments whose 3′ end is generated by MIWI/MILI slicing, while the 5′ end is created by an unknown nuclease that shapes it according to the footprint of the targeting PIWI protein (Fig. 1E). Recombinant MIWI/piRNA complex remains bound to its cleavage products, thereby protecting the two cleaved ends generated by slicer activity (Arif et al. 2022). It is also shown that for similar target-small RNA pairing configurations, sponge and Silkworm PIWI remain bound to their targets much longer than AGO clade Argonautes (Anzelon et al. 2021). We speculate that 5′ → 3′ exonuclease activity like that of XRN1 may act on such a stalled postcatalytic complex, generating the 5′ monophosphate end of the by-product fragment. Taken together, pachytene piRNAs are perfectly complementary to a target mRNA guide slicing in vivo, but the cleavage fragments remain bound to the PIWI complex.

### Unexpected MILI/MIWI slicer activity on targets with cleavage site mismatches

**FIGURE 2.**
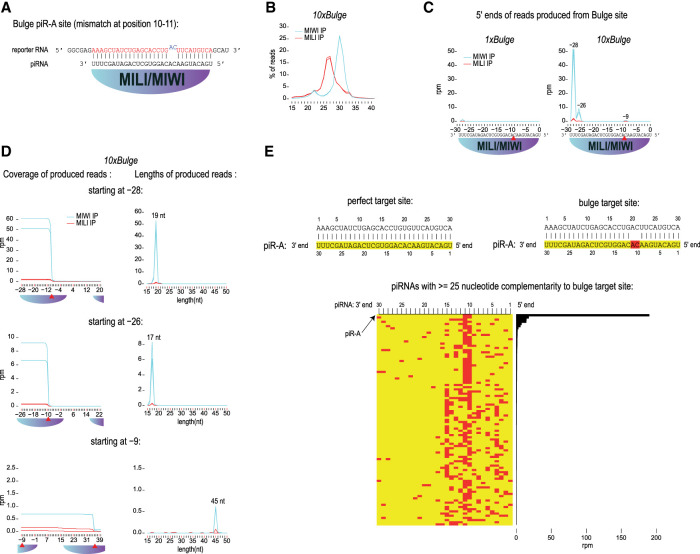
RNA targets with cleavage-site mismatches are sliced by PIWI proteins in vivo. (*A*) The bulge piR-A sites were designed to contain noncomplementary bases to piR-A nucleotides at positions 10 and 11. (*B*) Length profiles of immunoprecipitated small RNAs from *10xBulge* mouse. (*C*) The position (=distance from 5′ nt of targeting piR-A) and abundance of 5′ ends of reads produced from reporter target sites. In the case of *10xBulge*, the metaplot summarizes the read counts from all ten sites. Red triangle refers to expected position of MILI/MIWI cleavage guided by piR-A. (*D*) Coverage of reads is shown separately for RNAs with their 5′ end starting at a specific distance from the first piR-A target nucleotide (=0 distance). These RNAs are of specific lengths, with the 3′ ends always created by MILI/MIWI slicing at a subsequent site. (*E*) Complementary piRNAs potentially targeting the bulge site. The heatmap depicts position of mismatches (in red) between the targeting piRNA and the target site sequence, while the barplot shows the abundance of such piRNAs.

Immunopurified endogenous MIWI fails to slice a target RNA when a 2 nt mismatch (at positions 10 and 11 of the piRNA) exists between the guiding piRNA and the cleavage site (Reuter et al. 2011). Surprisingly, mapping of the sequencing reads from MIWI and MILI complexes isolated from the *10xBulge* knock-in mice revealed the tell-tale signatures of slicer activity (Fig. 2A–C). First, the bulged target is cleaved after the 10th nucleotide of the targeting piRNA, as indicated by the presence of the pre-piRNA fragment with the 5′ nucleotide at a distance −9 from the first nucleotide of the piRNA (Fig. 2C). Second, the 17 and 19 nt by-product fragments are also detected with a common 3′ end at the site of slicer cleavage (Fig. 2D). We examined whether any of the other thousands of pachytene piRNAs can perfectly pair with the mutated binding site but found none (Fig. 2E). This suggests that mismatches at the cleavage site are not inhibitory for slicing of a target RNA by a paired piRNA/PIWI complex in mouse testes. This in vivo observation is in direct contradiction to the piRNA targeting rules previously established for MIWI by in vitro slicer assays (Reuter et al. 2011). This is also very different to the situation with AGO clade Argonautes which are unable to cleave a target with a central mismatch both in vitro and in vivo (Meister 2013).

As in *10xPerf*, the pre-piRNA fragment originating from *10xBulge* has a length of 45 nt consistent with its 5′ and 3′ ends being generated by slicer activity from two consecutive bulged piRNA-targeting sites (Figs. 1G, 2D). This observation again reinforces the fact that slicer activity by MIWI and MILI fails to initiate generation of new piRNAs from the targets we examined. The absence of U1 as the 5′ nucleotide (which is a cytidine here) (Figs. 1G, 2) could be the reason for failed entry of the pre-piRNA into the piRNA biogenesis pathway (Kawaoka et al. 2011; Genzor et al. 2021) or it just shows that not all RNA targets of PIWI slicing enter the piRNA biogenesis pathway.

Immunopurified MIWI from mouse testes (Reuter et al. 2011) or a recombinant MIWI/piRNA complex (Arif et al. 2022) is shown to slice RNA targets when the guiding piRNA has a minimum of 21 nt complementarity (positions 2–22 of the piRNA). In vivo studies with mice expressing human pachytene piRNAs showed that signatures of target slicing were observed even when some mismatches (at positions 12–21) existed between the guiding piRNA and the target, as long as guide-target pairing was maintained between positions 2–11 (Goh et al. 2015). Recombinant sponge PIWI/guide RNA complex is also shown to require extensive complementarity (positions 2–16 of the guide) between the 25 nt guide RNA and target for slicing (Anzelon et al. 2021). In fact, increasing the pairing toward the 3′ end (from positions 2–16 to 2–21 of the guide) enhances the slicing activity of sponge PIWI, with no more than 2 nt mismatches (within positions 11–18) tolerated for efficient cleavage (Anzelon et al. 2021). Recent studies indicate that PIWI proteins on their own are inefficient slicers, with the zinc-finger protein GTSF1 being able to enhance the rate of slicing in vitro (Arif et al. 2022). Interestingly, GTSF1 can also reduce the requirement for extensive complementarity by promoting MIWI slicing with just 2–16 nt of the piRNA being paired with the target (Arif et al. 2022). Finally, a recent preprint study reports that GTSF1 can further relax piRNA/MIWI targeting rules, with even targets having a central mismatch being cleaved (Gainetdinov et al. 2022). Taken together, using the four knock-in mouse models we have uncovered important aspects of in vivo PIWI slicer activity that were not previously appreciated, and demonstrated that PIWI slicing rules in vivo are different from those currently established in vitro. The ability of accessory factors to modulate PIWI slicer activity, potentially via affecting PIWI structure, likely explains this deviation in vivo.

## MATERIALS AND METHODS

### Animal work

The transgenic Mouse Facility of the University of Geneva was used to generate the four knock-in mouse lines utilized in this project. This study was approved by the Canton of Geneva (GE-162-19).

### Generation of knock-in mouse lines

Knock-in mice were created by inserting one or ten complementary binding sites for a piRNA (piR-A) in the 3′ UTR of the *Ythdc2* gene (MGI: 2448561; NCBI Gene: 240255). Binding sites are perfectly complementary (*1xPerf* or *10xPerf*) or contain two mismatches (*1xBulge* or *10xBulge*) on the 10th and 11th position relative to the guide RNA. To create these reporter mouse lines, the *Ythdc2* locus of interest was targeted in single cell mouse embryos of the B6D2F1/J hybrid line (also called B6D2; The Jackson Laboratory, stock no. 100006).

Targeting of the locus was achieved by injecting two different guide RNAs (gRNAs from Integrated DNA Technologies) complexed with the Cas9 protein. The first gRNA (CAUAACGCUCUUCCAUAGCCGUUUUAGAGCUAUGCU) targeted the reverse strand at position chr18: 44887792. The second gRNA (GUGCAUUCUGUAGCUACAACGUUUUAGAGCUAUGCU) targeted the reverse strand at position chr18: 44887815. Combination of these two gRNAs and Cas9 endonuclease created a deletion of 23 bp in the last exon (exon 30) of *Ythdc2*. Two ssDNA oligonucleotide templates (Integrated DNA Technologies; 195 bp) containing the required single piRNA binding sites were coinjected. The two multiple binding sites reporter alleles were created by separately coinjecting two pUC57-Kan plasmids containing 10 piR-A binding sites (perfect or bulge) separated by spacers and flanked by homology arms to the *Ythdc2* locus of interest (Supplemental Fig. 2). The gRNAs were composed of two parts: one tracrRNA and one crRNA (sequences as above). The tracrRNA is 67 bases long (Integrated DNA Technologies, Cat. No. 1072533) and is the common part of the guide RNA that binds the Cas9. A mixture of 200 pmol of tracrRNA and crRNA were annealed by incubating 5 min at 95°C in 1xTE buffer, pH 7.5 (Integrated DNA Technologies; Cat. No. 11-05-01-05) and cooling down at room temperature for 30 min. The final volume was 10 µL. Annealed gRNAs were stored at −20°C and then used for mouse single cell embryo injection.

The single cell embryo injection mix was prepared right before the injection. Cas9 protein at 30 ng/µL final concentration (Integrated DNA Technologies; Cat. No. 1081058) was mixed with the annealed gRNAs at 0.6 pmol/µL final concentration. Template DNA was also added to the mix at 20 ng/µL final concentration and the volume was adjusted to 100 µL with TE buffer (pH 7.5). Complex formation was achieved by incubating the mix 10 min at room temperature. Finally, the mix was centrifuged at 13,000 rpm for 5 min at 4°C, and 50 µL of supernatant were collected and placed on ice.

### Genotyping

Primers (Forward: CTCAGGTTGGGGAACAGTTG, Reverse: ACTCTATGCCTCAAATCCACTC A) flanking the targeted insertion region in the mouse *Ythdc2* gene were used to genotype the knock-in alleles. The expected PCR product sizes were 281 bp for WT, 320 bp for *1xPerf/1xBulge* and 716 bp for *10xPerf/10xBulge*. PCR reactions were run with Phire Green Hot Start II PCR Master Mix (Thermo) under the following conditions: 98°C for 30 sec, 35 cycles of (98°C for 5 sec, 63°C for 5 sec and 72°C for 10 sec), 72°C for 60 sec, and lastly at 4°C to hold the reaction.

### PIWI immunoprecipitations

Immunoprecipitation from adult mouse testes was conducted as previously reported using anti-MILI and anti-MIWI rabbit polyclonal antibodies (Reuter et al. 2011). MILI- and MIWI-loaded piRNAs were extracted from the bead complexes by phenol–chloroform extraction, after proteinase K treatment.

### RNA library preparation and sequencing

piRNA libraries were prepared with NEBNext Multiplex Small RNA Library Prep Set for Illumina (New England Biolabs, Ref. E7300L) and sequenced at EMBL Genomic Core Facilities in Heidelberg using the NextSeq 500 platform (Illumina).

Total RNA extracted from adult whole testes was used as starting material for the testicular transcriptome sequencing. The Stranded Total RNA Ribo-Zero Plus kit from Illumina was used for the library preparation with 500 ng of total RNA as input. Library molarity and quality were assessed with the Qubit and Tapestation (DNA High sensitivity chip). Libraries were sequenced on a NovaSeq 6000 Illumina sequencer for SR100 reads (iGE3 Genomics Platform, University of Geneva).

### Quantification and statistical analysis of small RNA sequencing

Reads were sorted into individual libraries based on the barcodes, and the 3′ adapter sequences were clipped from the reads using cutadapt (Martin 2011) with only sequences of at least 15 nt left for further analysis (cutadapt parameters: -a AGATCGGAAGAGCACACGTCT -m 15 -e 0.2 -O 4 -q 10 –match-read-wildcards). The reads from small RNA-seq libraries were mapped to the mouse genome (GRCm38: Ensembl release 95) using STAR (Dobin et al. 2013) (with parameters: –runThreadN 10 -outFilterType BySJout –limitOutSJcollapsed 50000000 –limitIObufferSize 1500000000) and length distributions of the reads were plotted. Read counts were normalized to library sizes.

To see whether any piRNAs are produced from the reporters, the reads were mapped by bowtie (Langmead et al. 2009) (with parameters: -v 0 -a –best –strata) to the *Ythdc2* transcript sequence containing individual reporters: *1xPerf, 10xPerf, 1xBulge* and *10xBulge* (Supplemental Fig. 2). We calculated 5′ end and body coverages of the reads produced from individual reporters as well as coverages targeting the reporters. Counts of reads were divided by number of mapped sites and normalized to library sizes (rpm—reads per million). To characterize the overall production of piRNAs from the ten sites of *10xPerf* and *10xBulge* reporters, we also created the metaplots where we summarized the counts of piRNAs produced from individual positions of all ten target sites. The positions refer to the distance from the 5′ end of targeting piR-A piRNA. Therefore, nucleotide at position 0 refers to the nucleotide which pairs with the first nucleotide of piR-A. The position −9 refers to the nucleotide pairing with 10th piR-A nucleotide and is the position of the 5′ end of secondary piRNAs created by MILI or MIWI cleavage.

### Quantification and statistical analysis of long RNA sequencing

The reads from long RNA-seq libraries were mapped to the mouse genome (GRCm39 - Ensembl release 104) using salmon (Patro et al. 2017) (salmon quant with options -1 A –validateMappings–gcBias). Further analysis was performed using R (R Core Team 2017) and Bioconductor (Huber et al. 2015). The DESeq2 (Love et al. 2014) package was used to obtain normalized read counts for individual genes. The Volcano plots were plotted using the EnhancedVolcano function from the EnhancedVolcano 1.3.5 package (https://github.com/kevinblighe/EnhancedVolcano). To visualize expression of dysregulated genes across spermatogenic populations (NCBI: PRJNA317251), we calculated the *z*-scores of log_2_ expression (reads per million) for each of those genes and plotted the boxplots for individual samples. Similarly, the heatmap of *z*-scores was plotted using pheatmap 1.0.12.

## DATA DEPOSITION

The sequencing data generated in the study are deposited with the Gene Expression Omnibus (GEO: GSE219200).

## SUPPLEMENTAL MATERIAL

Supplemental material is available for this article.

## Supplementary Material

Supplemental Material
