# Supplementary material for: In vivo PIWI slicing in mouse testes deviates from rules established in vitro
Source: RNA. 2023 Mar;29(3):308–16. doi: 10.1261/rna.079349.122 (PMC9945443; doi:10.1261/rna.079349.122)
Supplement: Supplemental Material [file supp_29_3_308__DC1.html]

In vivo PIWI slicing in mouse testes deviates from rules established in vitro — Supplemental Material 

# In vivo PIWI slicing in mouse testes deviates from rules established in vitro

## Supplemental Material

- Supplemental\_Figure\_1.pdf
- Supplemental\_Figure\_2.pdf
- Supplemental\_Figure\_Legends.docx
