# Supplementary material for: In vivo PIWI slicing in mouse testes deviates from rules established in vitro
Source: RNA. 2023 Mar;29(3):308–16. doi: 10.1261/rna.079349.122 (PMC9945443; doi:10.1261/rna.079349.122)
Supplement: Supplemental Material [file supp_079349.122_Supplemental_Figure_1.pdf]

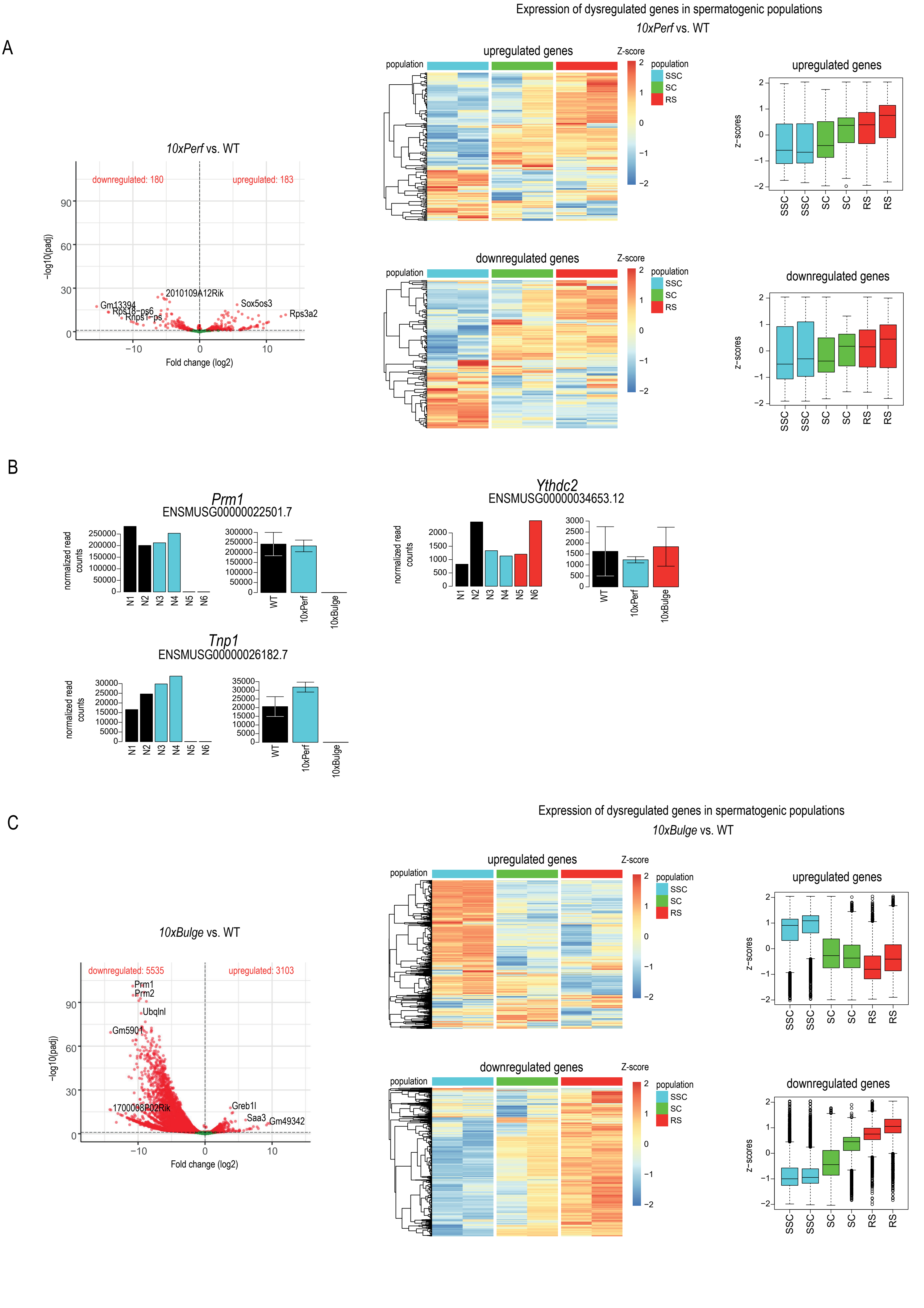

Supplemental Figure-1
