# Supplementary material for: In vivo PIWI slicing in mouse testes deviates from rules established in vitro
Source: RNA. 2023 Mar;29(3):308–16. doi: 10.1261/rna.079349.122 (PMC9945443; doi:10.1261/rna.079349.122)
Supplement: Supplemental Material [file supp_079349.122_Supplemental_Figure_2.pdf]

Supplemental Figure-2. The *Ythdc2* reporter sequences.

>1xPerf

GAGACAGGGTTTAACTCTTGTAGCCTGAGCTGTTCTAGAACTCACTGGGTAGACCGGGCTGGCATCAAACGTGGCCTCTCAAGTTCTGGGATTAAAGGCGTTTGTCACCACCGCCTCCCCAACTCATGA
GAGGTTCCTCTTTATACTTAATCTTGCCACAAAACAGAACTATTCTCATCGCTATGCGTTCCACATCCACGGTGGGCTCTTCCCTAAAACTGTGAGCACTTCAGTAACTATCATGTAAGCATTTCATTT
TGGGGACGATCATTACTCTTTTGGGTTGCGAGGTAAAAGAGTTAAATCTTTATTTCGGGTAATCGAGTTTTCAATGTGTCTCAGAATGAAAGATATATACTAAAAGGTTATGCGTTATTTAAAATCAAA
CCAGTCGGTCCCCTCACTGTACTTACTTTGCTCCTACTAGACCACGAACCCCAGAATTGCGGGGAAAACCCTTGTTTCCAGCGCGTTCGAGTTTGTGACTCTAGCGCTGGGCAGAAGAAGCCCGTTATA
AAGAAAGGAGCGAAGCATTAGATGATCGAGTGGGTAACCGAAATAATATGTCCTCCAATTAAATGACACTTTTGGAGAACCAAAGCCTCGGGGTCGTGAGGACCACAGCACCCCTCCAAGGACCGCATT
CCCCAGCGCAGCACCCGCACAGCCAGCGCTCCCGCGTTCTGGGGTCGCGCGCGGCGCGCCCTCGTGACGTCATCACGCGCGCGCCCGCCCGCCCGCCCGCAGCTTCTCTGGGTTAGAGTGGTGCCGGCG
CTTTCGCTTCCACCGCTGCCGTGAGCGGCGCTTTCCCGCCGGTCGCCCCCGGCGCTGTCTTCCGAGCGTTCAGTCCGCCGCGTCCTCACCGGCTCTCTTGTCTCTGTCACTGCTCGCTGGCAGCAGGCC
TGGCCGCTCCCACGCGGAGCGAGCTTCTCCTCAGGGCCATGTCCAGGCCGAGCAGCGTCTCGCCGCGTCCGCCGGCTCCTAGCGGAGGCGGCACGGGCGGCGGTGGAGGTGGCAGTGGCGGCGGCGGTG
GCGGTGGCGGCGGCGGCCCAGCTTCGTGTGGTCCTGGAGGCGGTGGCCGGGCCAAGGGCCTCAAAGACATTCGTATCGACGAGGAGGTGAAGATCGCCGTCAACATCGCTCTGGAGCGCTTCCGCTACG
GTGACCAGAGAGAAATGGAATTCCCTTCTTCTTTGACCAGTACGGAAAGAGCCTTTATTCACCGACTAAGTCAATCTCTTGGTTTAGTTTCTAAAAGTAAAGGAAAGGGAGCAAATAGATACCTAACTG
TGAAGAAGAAAGATGGATCAGAAACAGCTCATGCAATGATGACCTGTAATTTGACTCATAACACAAAACATGCTGTTAGGAGCCTAATTCAGAGATTTCCTGTCACCAATAAAGAACGTACTGAACTCC
TACCTAAAACAGAAAGAGGAAATGTGTTTGCAGTTGAAGCTGAAAACCGAGAAATGAGCAAGACAAGCGGGCGACTCAACAATGGCATTCCTCAAGTTCCTGTGAAAAGGGGAGAGTCTGAATTTGACT
CATTCAGGCAGTCTTTACCAGTTTTTGAGAAACAAGAAGAAATTGTTAAAATAATTAAGGAAAATAAAGTAGTTTTGATAGTAGGAGAAACTGGGTCTGGAAAGACCACACAGATTCCTCAGTTCCTGT
TAGATGATTGCTTTAAAAATGGCATCCCCTGCCGAATATTTTGTACTCAACCAAGACGACTAGCAGCGATTGCTGTGGCTGAAAGAGTTGCTGCAGAGAGAAGAGAAAGAATTGGTCAAACAATTGGTT
ATCAGATCCGATTAGAAAGCAGGGTTTCGCCAAAGACACTTCTGACATTTTGCACTAATGGGGTATTGCTTCGTACATTGATGGCAGGTGATAGCACATTGTCAACTGTGACCCATGTTATTGTGGATG
AAGTGCATGAAAGGGATCGATTCAGTGATTTTTTGCTTACTAAGTTAAGAGATTTGTTGCAAAAGCACCCAACTTTGAAACTAATTCTTTCGAGTGCTGCCTTGGATGTGAACCTCTTCATAAGATATT
TTGGAAGTTGTCCAGTGATATATATACAGGGAAGACCATTTGAAGTAAAAGAAATGTTTCTGGAAGATATTTTAAGAACTACAGGATATACCAACAAAGAAATGTTAAAGTACAAAAAGGAAAAGCAAC
GAGAGGAGAAACAACAGACCACCCTCACAGAGTGGTACTCAGCTCAAGAGAATACCTTCAAGCCTGAGTCTCAGAGGCAGAGAGCTGTTGCTAGTGTGTCTGAAGAGTATGACTTATTGGATGATGGAG
GTGATGCTGTCTTCAGTCAGCTGACAGAAAAAGATGTGAATTGCCTTGAACCATGGTTAATCAAGGAGATGGATGCCTGCCTTTCTGACATATGGCTGCATAAAGATGTTGATGCTTTTGCTCAGGTCT
TTCATCTTATTTTAACTGAAAATGTTAGTGTTGATTATAGGCATAGTGAGACCAGTGCAACAGCTCTGATGGTTGCTGCAGGACGGGGCTTTACGAGCCAAGTAGAGCAGTTAATTAGTATGGGAGCCA
ATGTCCACAGTAAAGCATCAAATGGCTGGATGGCTTTAGATTGGGCTAAGCACTTTGGACAGACTGAAATTGTGGATCTTCTAGAATCTTACAGTGCTTCATTGGAATTTGGAAATCTAGATGAAAGTT
CCTTGGTTCAGACAAATGGGAATGACCTCAGTGCAGAAGACAGAGAGCTGTTGAAAGCTTACCATCATAGTTTTGATGATGAGAAAGTAGACTTGGATTTGATCATGCATCTCCTATATAATATCTGCC
ATAGCTGTGATGCTGGTGCAATATTAATTTTTCTACCTGGATATGATGAAATTGTTGGTTTGAGGGATCGTATCCTCTTTGATGACAAGCGGTTTGCTGACAATACACATAGGTATCAAGTCTTTATGC
TTCATTCAAATATGCAGACATCTGATCAAAAGAAGGTATTAAAAAATCCACCTGCAGGTGTCCGAAAAATAATCCTTTCCACCAATATTGCTGAGACGAGCATCACTGTCAATGATGTTGTGTTTGTTA
TTGATTCTGGTAAGGTGAAAGAGAAATCCTTTGATGCACTGAATTTTGTTACAATGTTAAAAATGGTATGGATTTCCAAAGCTAGTGCAATACAGCGCAAAGGCAGAGCGGGACGGTGTAGACCTGGAA
TTTGTTTCCGTCTGTTTAGTAGACTTCGATTTCAGAACATGCTGGAATTTCAGACGCCAGAACTCTTGAGAATGCCATTGCAGGAACTTTGTTTACATACCAAGCTGTTAGCCCCAGTTAACTGCACCA
TTGCCGACTTCCTTATGAAAGCCCCTGAACCGCCTCCAGCTTTAATTGTGAGAAATGCTGTGCAGATGCTTAAGACAATAGATGCAATGGATGCATGGGAAGATCTGACTGAACTTGGGTATCATTTGG
CTGATTTGCCAGTAGAACCGCATCTTGGGAAAATGGTCCTGTGTGCAGTGGTTTTGAAGTGTCTGGACCCCATCCTTACAATTGCCTGCACGCTCGCTTACCGTGACCCTTTTGTCCTGCCTACCCAGG
CCTCTCAGAAACGGGCAGCTATGCTTTGTAGGAAACGCTTCACTGCAGGGACTTTCAGTGACCACATGGCACTTCTGAGAGCATTTCAGGCATGGCAGAAAGCACGAAGTGATGGGTGGGAGCGAGCCT
TTTGTGAAAAGAATTTTCTTTCACAAGCTACTATGGAAATCATTATAGGCATGAGAACACAGTTGCTTGGTCAGCTTAGAGCATCAGGTTTTGTTAGAGCACGAGGTGGTGGTGACATTCGAGATGTTA
ATACAAACTCCGAGAACTGGGCTGTTGTTAAAGCTGCATTGGTAGCAGGCATGTATCCTAATTTAGTCCACGTGGACAGAGAGAATGTAATATTGACAGGGCCAAAGGAAAAGAAAGTACGCTTTCATC
CCACTTCAGTTCTCAGTCAACCTCAATACAAAAAGATTCCTCCCGCCAATGGTCAGGCTGCAGCGATTCAGGCATTGCCCACAGATTGGCTTATTTATGATGAAATGACCAGAGCCCATCGAATTGCTA
ATATCAGATGTTGTTCAGCAGTGACACCTGTTACTGTCCTGGTGTTCTGTGGACCAGCAAGACTGGCAAGTAACGCTCTTCAGGAACCTTCATCCTTTAGAGCGGATGGTATTCCCAATGACAGCAGTG
ACAGTGAGATGGAAGACAGAACTACTGCCAATTTGGCTGCTTTGAAGCTTGATGAATGGCTTAACTTCAAACTAGAGCCAGAGGCTGCCAGTTTATTGCTACAGCTCAGACAGAAGTGGCATAGTTTGT
TTTTACGCCGAATGAGAGCGCCATCTAAACCTTGGTCTCAAGTCGATGAAGCTACCATACGAGCAATTATAGCTGTTTTAAGCACTGAAGAACAGTCTGCAGGTTTACAGCAGCCATCTGGAATTGGCC
AGCGGCCAAGACCCATGTCTTCAGAAGAACTTCCTTTGGCTTCATCTTGGAGATCCAATAATAGTAGGAAAAGTACAGCTGACACTGAGTTTGCGGATGGATCTACTACTGGAGAAAGAGTACTGATGA
AATCTCCATCTCCAGCCTTACATCCACCTCAGAAGTACAAAGATAGAGGAATTTTACATCCTAAGCGGAGCACCGATGACCGGTCCGATCAATCATCTGTGAAATCCACAGACAGCAGCAGTTACCCAA
GTCCTTGTGCCAGCCCCTCTCCTCCATCCTCAGGGAAGGGATCAAAATCTCCTTCACCAAGACCAAACATGCCTATTCGTTACTTCATAATGAAGAGTAGCAATCTGAGAAACCTTGAAATTTCTCAAC
AGAAGGGTATCTGGTCTACAACCCCTAGTAATGAGCGGAAGCTAAATCGAGCGTTTTGGGAGAGCAGCATGGTTTACTTGGTATTTTCTGTTCAAGGATCTGGACATTTCCAGGGATTTTCTAGGATGT
CTTCTGAGATAGGAAGGGAGAAGAGCCAGGACTGGGGGTCAGCTGGACTAGGTGGAGTATTTAAAGTGGAGTGGATACGCAAAGAAAGCCTTCCCTTTCAGTTTGCACACCATTTACTCAATCCTTGGA
ACGACAACAAGAAAGTACAGATAAGCAGAGATGGGCAGGAGCTAGAACCTCAGGTTGGGGAACAGTTGCTCCAGTTATGGGAACGCCTTCCATTGGGAGAAAAAACAACTTCTGATTGACATTCAGAAC
TCCTGGCTATGGAAGAGCGTTATGCCTGGCGAGAAAGCTATCTGAGCACCTGTGTTCATGTCAGCATGTTGTAGCTACAGAATGCACTGATTTTCTGAAAACCTTGATGAAGTGTTGTATGCCATGAGT
GGATTTGAGGCATAGAGTATTCCTTCAGAATCATCATCTTCATATAGAGGAAAAGATAATTTAAATTTTTCTAGTGGTTTTAAGGAAATTATTATATAATGTTTGTTAATTATCTGTTTCAGATGAACA
AAATAGCAATTTTGGTATACAACAGCACTTAATTTAATTTAATTTGAATGTAAACATTTCTTTAAAATATTCAGTTAATGACTGTTGATGCCAAAGTCTTTTCTTTTTGCTCTTTCCAGACAGGGTTTC
TTTCTTTGTTTAGCCTCAACTGTCCAGGAACTAGCTCTGTAAGACCAGGCTGACCTCAAACTCACAGATATCTGCCTGTTTGTTTCTGCCTCCTGAATACTGGAATTAAAGGCTTGCACCACCTCTGCC
TGAGATGCCAAACTCTTATTGTTGACTTTTTGGTTGTGAGCCTATCCTTTAATGGCTGAGCCATCTCTCCAGCCCTAATGTTGACTTTTTAATTCAGGAAAGGCATAAATATATCAGTACATCTAAGAA
CCATTCTAGAAAAGTCGGTTCTTAGGTTTATCATTAAAAAAACAACCAACACACTAAAATTTTAAATTATCTCTTACAGAACTTGTATACATAAGGGCTATTCATTGTTATTTTTAGCCTGAAATATAG
TAAAAAACAGGAGTGCTATTTTTCTCTGATTACAGAATGAGATAAAAATCTCAAGAAAGGTAATTTCTCTTGACTTTTGATGAGATCAGTGCTTAATTTCATTCCTAGTCCTGTCTCTTTTAACCTTAT
GTAAAATATACCCATTCTTTTTTTGCTTCCCATGAAGCAACCTTCACATAACTATTACTATTATGACCTCTTTGTTTTGCTACAAGAACCCAATTTAAAATAACAAAAATTTATGGAAATTAATACAAG
TTTTAAAAAATGCTCAGCGGTTATTGATAGTTTGGTTGCTATATATCTTTGAAACTAAAGCTCACACAACCCATAATTCTTCATGTCTTTTTTCATTATTTTTTAATCTTTTTCTTTCACCTATTATAT
GTAAATGGGGCATTTATTTTTCTAAGTCTGTGTTAATGCTAAGTCTCATGTCTCTTTACCATCAGAGAGTTACTTTCTTTTATGCTTTTACCACCATTGTTTACAGGTTTTCTTTGAGACTCTTGGAAG
ATAAAGTAGAAGTGCAGGGCAGAACATCAGACATATCTTTGCTACTGAGCTCTAAATAGAGCAGGCTTGATAGTACTTTTCATAGCATTTGAAAATGAATTTAATTTAAAGATGAAAAGAAAACAAATG
TCTGGTTCAGTATTTGAGGGCAAAGATATAGAAAATCATGGATTGGCATGAAGTCTTGAGAACTCTTCTACTTTCTCTGTGAAAATGTATTAATGCATTGTTTAAAGATATCAGCTATAACTTTTGCCT
GTATTAGAAATTGTGATATATATATATGTATATATGTGTATATACATATAGATACACACACATATATGTATATATATATGATACATGAATGCATATATGTATACACACACTCACACACTCATTCATAAACT
TTGAAGCTGAGCCAGAGTGAATTATGCTTCATTTATGTGTATTACATTTCTTTTGAAACTTACAATTTTCCTGATAATGTATACTTTTTTAAGGTATATAAGTAGAGGTTTTCAGCATTCTAAATTTGG
AAGAACTTTTAAAGAAATAAAATTTGTTTAAATGTTTCTTGTTTTGTAATATCTAGACTTGTAAATAAATGATGGATTTGTTGGTAGAGTGGATATAATTTGAATGTAATAAAAATACTGTTGAATAAA
TGAATTTTAAATATACTTATCAAATGAAATA

>1xBulge

GAGACAGGGTTTAACTCTTGTAGCCTGAGCTGTTCTAGAACTCACTGGGTAGACCGGGCTGGCATCAAACGTGGCCTCTCAAGTTCTGGGATTAAAGGCGTTTGTCACCACCGCCTCCCCAACTCATGA
GAGGTTCCTCTTTATACTTAATCTTGCCACAAAACAGAACTATTCTCATCGCTATGCGTTCCACATCCACGGTGGGCTCTTCCCTAAAACTGTGAGCACTTCAGTAACTATCATGTAAGCATTTCATTT
TGGGGACGATCATTACTCTTTTGGGTTGCGAGGTAAAAGAGTTAAATCTTTATTTCGGGTAATCGAGTTTTCAATGTGTCTCAGAATGAAAGATATATACTAAAAGGTTATGCGTTATTTAAAATCAAA
CCAGTCGGTCCCCTCACTGTACTTACTTTGCTCCTACTAGACCACGAACCCCAGAATTGCGGGGAAAACCCTTGTTTCCAGCGCGTTCGAGTTTGTGACTCTAGCGCTGGGCAGAAGAAGCCCGTTATA
AAGAAAGGAGCGAAGCATTAGATGATCGAGTGGGTAACCGAAATAATATGTCCTCCAATTAAATGACACTTTTGGAGAACCAAAGCCTCGGGGTCGTGAGGACCACAGCACCCCTCCAAGGACCGCATT
CCCCAGCGCAGCACCCGCACAGCCAGCGCTCCCGCGTTCTGGGGTCGCGCGCGGCGCGCCCTCGTGACGTCATCACGCGCGCGCCCGCCCGCCCGCCCGCAGCTTCTCTGGGTTAGAGTGGTGCCGGCG
CTTTCGCTTCCACCGCTGCCGTGAGCGGCGCTTTCCCGCCGGTCGCCCCCGGCGCTGTCTTCCGAGCGTTCAGTCCGCCGCGTCCTCACCGGCTCTCTTGTCTCTGTCACTGCTCGCTGGCAGCAGGCC
TGGCCGCTCCCACGCGGAGCGAGCTTCTCCTCAGGGCCATGTCCAGGCCGAGCAGCGTCTCGCCGCGTCCGCCGGCTCCTAGCGGAGGCGGCACGGGCGGCGGTGGAGGTGGCAGTGGCGGCGGCGGTG
GCGGTGGCGGCGGCGGCCCAGCTTCGTGTGGTCCTGGAGGCGGTGGCCGGGCCAAGGGCCTCAAAGACATTCGTATCGACGAGGAGGTGAAGATCGCCGTCAACATCGCTCTGGAGCGCTTCCGCTACG
GTGACCAGAGAGAAATGGAATTCCCTTCTTCTTTGACCAGTACGGAAAGAGCCTTTATTCACCGACTAAGTCAATCTCTTGGTTTAGTTTCTAAAAGTAAAGGAAAGGGAGCAAATAGATACCTAACTG
TGAAGAAGAAAGATGGATCAGAAACAGCTCATGCAATGATGACCTGTAATTTGACTCATAACACAAAACATGCTGTTAGGAGCCTAATTCAGAGATTTCCTGTCACCAATAAAGAACGTACTGAACTCC
TACCTAAAACAGAAAGAGGAAATGTGTTTGCAGTTGAAGCTGAAAACCGAGAAATGAGCAAGACAAGCGGGCGACTCAACAATGGCATTCCTCAAGTTCCTGTGAAAAGGGGAGAGTCTGAATTTGACT
CATTCAGGCAGTCTTTACCAGTTTTTGAGAAACAAGAAGAAATTGTTAAAATAATTAAGGAAAATAAAGTAGTTTTGATAGTAGGAGAAACTGGGTCTGGAAAGACCACACAGATTCCTCAGTTCCTGT
TAGATGATTGCTTTAAAAATGGCATCCCCTGCCGAATATTTTGTACTCAACCAAGACGACTAGCAGCGATTGCTGTGGCTGAAAGAGTTGCTGCAGAGAGAAGAGAAAGAATTGGTCAAACAATTGGTT
ATCAGATCCGATTAGAAAGCAGGGTTTCGCCAAAGACACTTCTGACATTTTGCACTAATGGGGTATTGCTTCGTACATTGATGGCAGGTGATAGCACATTGTCAACTGTGACCCATGTTATTGTGGATG
AAGTGCATGAAAGGGATCGATTCAGTGATTTTTTGCTTACTAAGTTAAGAGATTTGTTGCAAAAGCACCCAACTTTGAAACTAATTCTTTCGAGTGCTGCCTTGGATGTGAACCTCTTCATAAGATATT
TTGGAAGTTGTCCAGTGATATATATACAGGGAAGACCATTTGAAGTAAAAGAAATGTTTCTGGAAGATATTTTAAGAACTACAGGATATACCAACAAAGAAATGTTAAAGTACAAAAAGGAAAAGCAAC
GAGAGGAGAAACAACAGACCACCCTCACAGAGTGGTACTCAGCTCAAGAGAATACCTTCAAGCCTGAGTCTCAGAGGCAGAGAGCTGTTGCTAGTGTGTCTGAAGAGTATGACTTATTGGATGATGGAG
GTGATGCTGTCTTCAGTCAGCTGACAGAAAAAGATGTGAATTGCCTTGAACCATGGTTAATCAAGGAGATGGATGCCTGCCTTTCTGACATATGGCTGCATAAAGATGTTGATGCTTTTGCTCAGGTCT
TTCATCTTATTTTAACTGAAAATGTTAGTGTTGATTATAGGCATAGTGAGACCAGTGCAACAGCTCTGATGGTTGCTGCAGGACGGGGCTTTACGAGCCAAGTAGAGCAGTTAATTAGTATGGGAGCCA
ATGTCCACAGTAAAGCATCAAATGGCTGGATGGCTTTAGATTGGGCTAAGCACTTTGGACAGACTGAAATTGTGGATCTTCTAGAATCTTACAGTGCTTCATTGGAATTTGGAAATCTAGATGAAAGTT

CCTTGGTTCAGACAAATGGGAATGACCTCAGTGCAGAAGACAGAGAGCTGTTGAAAGCTTACCATCATAGTTTTGATGATGAGAAAGTAGACTTGGATTTGATCATGCATCTCCTATATAATATCTGCC
ATAGCTGTGATGCTGGTGCAATATTAATTTTTCTACCTGGATATGATGAAATTGTTGGTTTGAGGGATCGTATCCTCTTTGATGACAAGCGGTTTGCTGACAATACACATAGGTATCAAGTCTTTATGC
TTCATTCAAATATGCAGACATCTGATCAAAAGAAGGTATTAAAAAATCCACCTGCAGGTGTCCGAAAAATAATCCTTTCCACCAATATTGCTGAGACGAGCATCACTGTCAATGATGTTGTGTTTGTTA
TTGATTCTGGTAAGGTGAAAGAGAAATCCTTTGATGCACTGAATTTTGTTACAATGTTAAAAATGGTATGGATTTCCAAAGCTAGTGCAATACAGCGCAAAGGCAGAGCGGGACGGTGTAGACCTGGAA
TTTGTTTCCGTCTGTTTAGTAGACTTCGATTTCAGAACATGCTGGAATTTCAGACGCCAGAACTCTTGAGAATGCCATTGCAGGAACTTTGTTTACATACCAAGCTGTTAGCCCCAGTTAACTGCACCA
TTGCCGACTTCCTTATGAAAGCCCCTGAACCGCCTCCAGCTTTAATTGTGAGAAATGCTGTGCAGATGCTTAAGACAATAGATGCAATGGATGCATGGGAAGATCTGACTGAACTTGGGTATCATTTGG
CTGATTTGCCAGTAGAACCGCATCTTGGGAAAATGGTCCTGTGTGCAGTGGTTTTGAAGTGTCTGGACCCCATCCTTACAATTGCCTGCACGCTCGCTTACCGTGACCCTTTTGTCCTGCCTACCCAGG
CCTCTCAGAAACGGGCAGCTATGCTTTGTAGGAAACGCTTCACTGCAGGGACTTTCAGTGACCACATGGCACTTCTGAGAGCATTTCAGGCATGGCAGAAAGCACGAAGTGATGGGTGGGAGCGAGCCT
TTTGTGAAAAGAATTTTCTTTCACAAGCTACTATGGAAATCATTATAGGCATGAGAACACAGTTGCTTGGTCAGCTTAGAGCATCAGGTTTTGTTAGAGCACGAGGTGGTGGTGACATTCGAGATGTTA
ATACAAACTCCGAGAACTGGGCTGTTGTTAAAGCTGCATTGGTAGCAGGCATGTATCCTAATTTAGTCCACGTGGACAGAGAGAATGTAATATTGACAGGGCCAAAGGAAAAGAAAGTACGCTTTCATC
CCACTTCAGTTCTCAGTCAACCTCAATACAAAAAGATTCCTCCCGCCAATGGTCAGGCTGCAGCGATTCAGGCATTGCCCACAGATTGGCTTATTTATGATGAAATGACCAGAGCCCATCGAATTGCTA
ATATCAGATGTTGTTCAGCAGTGACACCTGTTACTGTCCTGGTGTTCTGTGGACCAGCAAGACTGGCAAGTAACGCTCTTCAGGAACCTTCATCCTTTAGAGCGGATGGTATTCCCAATGACAGCAGTG
ACAGTGAGATGGAAGACAGAACTACTGCCAATTTGGCTGCTTTGAAGCTTGATGAATGGCTTAACTTCAAACTAGAGCCAGAGGCTGCCAGTTTATTGCTACAGCTCAGACAGAAGTGGCATAGTTTGT
TTTTACGCCGAATGAGAGCGCCATCTAAACCTTGGTCTCAAGTCGATGAAGCTACCATACGAGCAATTATAGCTGTTTTAAGCACTGAAGAACAGTCTGCAGGTTTACAGCAGCCATCTGGAATTGGCC
AGCGGCCAAGACCCATGTCTTCAGAAGAACTTCCTTTGGCTTCATCTTGGAGATCCAATAATAGTAGGAAAAGTACAGCTGACACTGAGTTTGCGGATGGATCTACTACTGGAGAAAGAGTACTGATGA
AATCTCCATCTCCAGCCTTACATCCACCTCAGAAGTACAAAGATAGAGGAATTTTACATCCTAAGCGGAGCACCGATGACCGGTCCGATCAATCATCTGTGAAATCCACAGACAGCAGCAGTTACCCAA
GTCCTTGTGCCAGCCCCTCTCCTCCATCCTCAGGGAAGGGATCAAAATCTCCTTCACCAAGACCAAACATGCCTATTCGTTACTTCATAATGAAGAGTAGCAATCTGAGAAACCTTGAAATTTCTCAAC
AGAAGGGTATCTGGTCTACAACCCCTAGTAATGAGCGGAAGCTAAATCGAGCGTTTTGGGAGAGCAGCATGGTTTACTTGGTATTTTCTGTTCAAGGATCTGGACATTTCCAGGGATTTTCTAGGATGT
CTTCTGAGATAGGAAGGGAGAAGAGCCAGGACTGGGGGTCAGCTGGACTAGGTGGAGTATTTAAAGTGGAGTGGATACGCAAAGAAAGCCTTCCCTTTCAGTTTGCACACCATTTACTCAATCCTTGGA
ACGACAACAAGAAAGTACAGATAAGCAGAGATGGGCAGGAGCTAGAACCTCAGGTTGGGGAACAGTTGCTCCAGTTATGGGAACGCCTTCCATTGGGAGAAAAAACAACTTCTGATTGACATTCAGAAC
TCCTGGCTATGGAAGAGCGTTATGCCTGGCGAGAAAGCTATCTGAGCACCTGACTTCATGTCAGCATGTTGTAGCTACAGAATGCACTGATTTTCTGAAAACCTTGATGAAGTGTTGTATGCCATGAGT
GGATTTGAGGCATAGAGTATTCCTTCAGAATCATCATCTTCATATAGAGGAAAAGATAATTTAAATTTTTCTAGTGGTTTTAAGGAAATTATTATATAATGTTTGTTAATTATCTGTTTCAGATGAACA
AAATAGCAATTTTGGTATACAACAGCACTTAATTTAATTTAATTTGAATGTAAACATTTCTTTAAAATATTCAGTTAATGACTGTTGATGCCAAAGTCTTTTCTTTTTGCTCTTTCCAGACAGGGTTTC
TTTCTTTGTTTAGCCTCAACTGTCCAGGAACTAGCTCTGTAAGACCAGGCTGACCTCAAACTCACAGATATCTGCCTGTTTGTTTCTGCCTCCTGAATACTGGAATTAAAGGCTTGCACCACCTCTGCC
TGAGATGCCAAACTCTTATTGTTGACTTTTTGGTTGTGAGCCTATCCTTTAATGGCTGAGCCATCTCTCCAGCCCTAATGTTGACTTTTTAATTCAGGAAAGGCATAAATATATCAGTACATCTAAGAA
CCATTCTAGAAAAGTCGGTTCTTAGGTTTATCATTAAAAAAACAACCAACACACTAAAATTTTAAATTATCTCTTACAGAACTTGTATACATAAGGGCTATTCATTGTTATTTTTAGCCTGAAATATAG
TAAAAAACAGGAGTGCTATTTTTCTCTGATTACAGAATGAGATAAAAATCTCAAGAAAGGTAATTTCTCTTGACTTTTGATGAGATCAGTGCTTAATTTCATTCCTAGTCCTGTCTCTTTTAACCTTAT
GTAAAATATACCCATTCTTTTTTTGCTTCCCATGAAGCAACCTTCACATAACTATTACTATTATGACCTCTTTGTTTTGCTACAAGAACCCAATTTAAAATAACAAAAATTTATGGAAATTAATACAAG
TTTTAAAAAATGCTCAGCGGTTATTGATAGTTTGGTTGCTATATATCTTTGAAACTAAAGCTCACACAACCCATAATTCTTCATGTCTTTTTTCATTATTTTTTAATCTTTTTCTTTCACCTATTATAT
GTAAATGGGGCATTTATTTTTCTAAGTCTGTGTTAATGCTAAGTCTCATGTCTCTTTACCATCAGAGAGTTACTTTCTTTTATGCTTTTACCACCATTGTTTACAGGTTTTCTTTGAGACTCTTGGAAG
ATAAAGTAGAAGTGCAGGGCAGAACATCAGACATATCTTTGCTACTGAGCTCTAAATAGAGCAGGCTTGATAGTACTTTTCATAGCATTTGAAAATGAATTTAATTTAAAGATGAAAAGAAAACAAATG
TCTGGTTCAGTATTTGAGGGCAAAGATATAGAAAATCATGGATTGGCATGAAGTCTTGAGAACTCTTCTACTTTCTCTGTGAAAATGTATTAATGCATTGTTTAAAGATATCAGCTATAACTTTTGCCT
GTATTAGAAATTGTGATATATATATGTATATATGTGTATATACATATAGATACACACACATATATGTATATATATATGATACATGAATGCATATATGTATACACACACTCACACACTCATTCATAAACT
TTGAAGCTGAGCCAGAGTGAATTATGCTTCATTTATGTGTATTACATTTCTTTTGAAACTTACAATTTTCCTGATAATGTATACTTTTTTAAGGTATATAAGTAGAGGTTTTCAGCATTCTAAATTTGG
AAGAACTTTTAAAGAAATAAAATTTGTTTAAATGTTTCTTGTTTTGTAATATCTAGACTTGTAAATAAATGATGGATTTGTTGGTAGAGTGGATATAATTTGAATGTAATAAAAATACTGTTGAATAAA
TGAATTTTAAATATACTTATCAAATGAAATA

>10xPerf

GAGACAGGGTTTAACTCTTGTAGCCTGAGCTGTTCTAGAACTCACTGGGTAGACCGGGCTGGCATCAAACGTGGCCTCTCAAGTTCTGGGATTAAAGGCGTTTGTCACCACCGCCTCCCCAACTCATGA
GAGGTTCCTCTTTATACTTAATCTTGCCACAAAACAGAACTATTCTCATCGCTATGCGTTCCACATCCACGGTGGGCTCTTCCCTAAAACTGTGAGCACTTCAGTAACTATCATGTAAGCATTTCATTT
TGGGGACGATCATTACTCTTTTGGGTTGCGAGGTAAAAGAGTTAAATCTTTATTTCGGGTAATCGAGTTTTCAATGTGTCTCAGAATGAAAGATATATACTAAAAGGTTATGCGTTATTTAAAATCAAA
CCAGTCGGTCCCCTCACTGTACTTACTTTGCTCCTACTAGACCACGAACCCCAGAATTGCGGGGAAAACCCTTGTTTCCAGCGCGTTCGAGTTTGTGACTCTAGCGCTGGGCAGAAGAAGCCCGTTATA
AAGAAAGGAGCGAAGCATTAGATGATCGAGTGGGTAACCGAAATAATATGTCCTCCAATTAAATGACACTTTTGGAGAACCAAAGCCTCGGGGTCGTGAGGACCACAGCACCCCTCCAAGGACCGCATT
CCCCAGCGCAGCACCCGCACAGCCAGCGCTCCCGCGTTCTGGGGTCGCGCGCGGCGCGCCCTCGTGACGTCATCACGCGCGCGCCCGCCCGCCCGCCCGCAGCTTCTCTGGGTTAGAGTGGTGCCGGCG
CTTTCGCTTCCACCGCTGCCGTGAGCGGCGCTTTCCCGCCGGTCGCCCCCGGCGCTGTCTTCCGAGCGTTCAGTCCGCCGCGTCCTCACCGGCTCTCTTGTCTCTGTCACTGCTCGCTGGCAGCAGGCC
TGGCCGCTCCCACGCGGAGCGAGCTTCTCCTCAGGGCCATGTCCAGGCCGAGCAGCGTCTCGCCGCGTCCGCCGGCTCCTAGCGGAGGCGGCACGGGCGGCGGTGGAGGTGGCAGTGGCGGCGGCGGTG
GCGGTGGCGGCGGCGGCCCAGCTTCGTGTGGTCCTGGAGGCGGTGGCCGGGCCAAGGGCCTCAAAGACATTCGTATCGACGAGGAGGTGAAGATCGCCGTCAACATCGCTCTGGAGCGCTTCCGCTACG
GTGACCAGAGAGAAATGGAATTCCCTTCTTCTTTGACCAGTACGGAAAGAGCCTTTATTCACCGACTAAGTCAATCTCTTGGTTTAGTTTCTAAAAGTAAAGGAAAGGGAGCAAATAGATACCTAACTG
TGAAGAAGAAAGATGGATCAGAAACAGCTCATGCAATGATGACCTGTAATTTGACTCATAACACAAAACATGCTGTTAGGAGCCTAATTCAGAGATTTCCTGTCACCAATAAAGAACGTACTGAACTCC
TACCTAAAACAGAAAGAGGAAATGTGTTTGCAGTTGAAGCTGAAAACCGAGAAATGAGCAAGACAAGCGGGCGACTCAACAATGGCATTCCTCAAGTTCCTGTGAAAAGGGGAGAGTCTGAATTTGACT
CATTCAGGCAGTCTTTACCAGTTTTTGAGAAACAAGAAGAAATTGTTAAAATAATTAAGGAAAATAAAGTAGTTTTGATAGTAGGAGAAACTGGGTCTGGAAAGACCACACAGATTCCTCAGTTCCTGT
TAGATGATTGCTTTAAAAATGGCATCCCCTGCCGAATATTTTGTACTCAACCAAGACGACTAGCAGCGATTGCTGTGGCTGAAAGAGTTGCTGCAGAGAGAAGAGAAAGAATTGGTCAAACAATTGGTT
ATCAGATCCGATTAGAAAGCAGGGTTTCGCCAAAGACACTTCTGACATTTTGCACTAATGGGGTATTGCTTCGTACATTGATGGCAGGTGATAGCACATTGTCAACTGTGACCCATGTTATTGTGGATG
AAGTGCATGAAAGGGATCGATTCAGTGATTTTTTGCTTACTAAGTTAAGAGATTTGTTGCAAAAGCACCCAACTTTGAAACTAATTCTTTCGAGTGCTGCCTTGGATGTGAACCTCTTCATAAGATATT
TTGGAAGTTGTCCAGTGATATATATACAGGGAAGACCATTTGAAGTAAAAGAAATGTTTCTGGAAGATATTTTAAGAACTACAGGATATACCAACAAAGAAATGTTAAAGTACAAAAAGGAAAAGCAAC
GAGAGGAGAAACAACAGACCACCCTCACAGAGTGGTACTCAGCTCAAGAGAATACCTTCAAGCCTGAGTCTCAGAGGCAGAGAGCTGTTGCTAGTGTGTCTGAAGAGTATGACTTATTGGATGATGGAG
GTGATGCTGTCTTCAGTCAGCTGACAGAAAAAGATGTGAATTGCCTTGAACCATGGTTAATCAAGGAGATGGATGCCTGCCTTTCTGACATATGGCTGCATAAAGATGTTGATGCTTTTGCTCAGGTCT
TTCATCTTATTTTAACTGAAAATGTTAGTGTTGATTATAGGCATAGTGAGACCAGTGCAACAGCTCTGATGGTTGCTGCAGGACGGGGCTTTACGAGCCAAGTAGAGCAGTTAATTAGTATGGGAGCCA
ATGTCCACAGTAAAGCATCAAATGGCTGGATGGCTTTAGATTGGGCTAAGCACTTTGGACAGACTGAAATTGTGGATCTTCTAGAATCTTACAGTGCTTCATTGGAATTTGGAAATCTAGATGAAAGTT
CCTTGGTTCAGACAAATGGGAATGACCTCAGTGCAGAAGACAGAGAGCTGTTGAAAGCTTACCATCATAGTTTTGATGATGAGAAAGTAGACTTGGATTTGATCATGCATCTCCTATATAATATCTGCC
ATAGCTGTGATGCTGGTGCAATATTAATTTTTCTACCTGGATATGATGAAATTGTTGGTTTGAGGGATCGTATCCTCTTTGATGACAAGCGGTTTGCTGACAATACACATAGGTATCAAGTCTTTATGC
TTCATTCAAATATGCAGACATCTGATCAAAAGAAGGTATTAAAAAATCCACCTGCAGGTGTCCGAAAAATAATCCTTTCCACCAATATTGCTGAGACGAGCATCACTGTCAATGATGTTGTGTTTGTTA
TTGATTCTGGTAAGGTGAAAGAGAAATCCTTTGATGCACTGAATTTTGTTACAATGTTAAAAATGGTATGGATTTCCAAAGCTAGTGCAATACAGCGCAAAGGCAGAGCGGGACGGTGTAGACCTGGAA
TTTGTTTCCGTCTGTTTAGTAGACTTCGATTTCAGAACATGCTGGAATTTCAGACGCCAGAACTCTTGAGAATGCCATTGCAGGAACTTTGTTTACATACCAAGCTGTTAGCCCCAGTTAACTGCACCA
TTGCCGACTTCCTTATGAAAGCCCCTGAACCGCCTCCAGCTTTAATTGTGAGAAATGCTGTGCAGATGCTTAAGACAATAGATGCAATGGATGCATGGGAAGATCTGACTGAACTTGGGTATCATTTGG
CTGATTTGCCAGTAGAACCGCATCTTGGGAAAATGGTCCTGTGTGCAGTGGTTTTGAAGTGTCTGGACCCCATCCTTACAATTGCCTGCACGCTCGCTTACCGTGACCCTTTTGTCCTGCCTACCCAGG
CCTCTCAGAAACGGGCAGCTATGCTTTGTAGGAAACGCTTCACTGCAGGGACTTTCAGTGACCACATGGCACTTCTGAGAGCATTTCAGGCATGGCAGAAAGCACGAAGTGATGGGTGGGAGCGAGCCT
TTTGTGAAAAGAATTTTCTTTCACAAGCTACTATGGAAATCATTATAGGCATGAGAACACAGTTGCTTGGTCAGCTTAGAGCATCAGGTTTTGTTAGAGCACGAGGTGGTGGTGACATTCGAGATGTTA
ATACAAACTCCGAGAACTGGGCTGTTGTTAAAGCTGCATTGGTAGCAGGCATGTATCCTAATTTAGTCCACGTGGACAGAGAGAATGTAATATTGACAGGGCCAAAGGAAAAGAAAGTACGCTTTCATC
CCACTTCAGTTCTCAGTCAACCTCAATACAAAAAGATTCCTCCCGCCAATGGTCAGGCTGCAGCGATTCAGGCATTGCCCACAGATTGGCTTATTTATGATGAAATGACCAGAGCCCATCGAATTGCTA
ATATCAGATGTTGTTCAGCAGTGACACCTGTTACTGTCCTGGTGTTCTGTGGACCAGCAAGACTGGCAAGTAACGCTCTTCAGGAACCTTCATCCTTTAGAGCGGATGGTATTCCCAATGACAGCAGTG
ACAGTGAGATGGAAGACAGAACTACTGCCAATTTGGCTGCTTTGAAGCTTGATGAATGGCTTAACTTCAAACTAGAGCCAGAGGCTGCCAGTTTATTGCTACAGCTCAGACAGAAGTGGCATAGTTTGT
TTTTACGCCGAATGAGAGCGCCATCTAAACCTTGGTCTCAAGTCGATGAAGCTACCATACGAGCAATTATAGCTGTTTTAAGCACTGAAGAACAGTCTGCAGGTTTACAGCAGCCATCTGGAATTGGCC
AGCGGCCAAGACCCATGTCTTCAGAAGAACTTCCTTTGGCTTCATCTTGGAGATCCAATAATAGTAGGAAAAGTACAGCTGACACTGAGTTTGCGGATGGATCTACTACTGGAGAAAGAGTACTGATGA
AATCTCCATCTCCAGCCTTACATCCACCTCAGAAGTACAAAGATAGAGGAATTTTACATCCTAAGCGGAGCACCGATGACCGGTCCGATCAATCATCTGTGAAATCCACAGACAGCAGCAGTTACCCAA
GTCCTTGTGCCAGCCCCTCTCCTCCATCCTCAGGGAAGGGATCAAAATCTCCTTCACCAAGACCAAACATGCCTATTCGTTACTTCATAATGAAGAGTAGCAATCTGAGAAACCTTGAAATTTCTCAAC
AGAAGGGTATCTGGTCTACAACCCCTAGTAATGAGCGGAAGCTAAATCGAGCGTTTTGGGAGAGCAGCATGGTTTACTTGGTATTTTCTGTTCAAGGATCTGGACATTTCCAGGGATTTTCTAGGATGT
CTTCTGAGATAGGAAGGGAGAAGAGCCAGGACTGGGGGTCAGCTGGACTAGGTGGAGTATTTAAAGTGGAGTGGATACGCAAAGAAAGCCTTCCCTTTCAGTTTGCACACCATTTACTCAATCCTTGGA
ACGACAACAAGAAAGTACAGATAAGCAGAGATGGGCAGGAGCTAGAACCTCAGGTTGGGGAACAGTTGCTCCAGTTATGGGAACGCCTTCCATTGGGAGAAAAAACAACTTCTGATTGACATTCAGAAC
TCCTGGCTATGGAAGAGCGTTATGCCTAAAGCTATCTGAGCACCTGTGTTCATGTCACGCCAGCTGGCGTAAAAAGCTATCTGAGCACCTGTGTTCATGTCATAGCGAAGAGGCCCGAAAGCTATCTGA
GCACCTGTGTTCATGTCACACCGATCGCCCTTCAAAGCTATCTGAGCACCTGTGTTCATGTCACCAACAGTTGCGCAGAAAGCTATCTGAGCACCTGTGTTCATGTCACCTGAAAGGCGAAAGAAAGCT
ATCTGAGCACCTGTGTTCATGTCAGCGCTTTGCCTGGTTAAAGCTATCTGAGCACCTGTGTTCATGTCATCCGGCACCAGAAGCAAAGCTATCTGAGCACCTGTGTTCATGTCAGGTGCCGGAAAGCTG
AAAGCTATCTGAGCACCTGTGTTCATGTCAGCTGGAGTGCGATCTAAAGCTATCTGAGCACCTGTGTTCATGTCAGTTGTAGCTACAGAATGCACTGATTTTCTGAAAACCTTGATGAAGTGTTGTATG
CCATGAGTGGATTTGAGGCATAGAGTATTCCTTCAGAATCATCATCTTCATATAGAGGAAAAGATAATTTAAATTTTTCTAGTGGTTTTAAGGAAATTATTATATAATGTTTGTTAATTATCTGTTTCA
GATGAACAAAATAGCAATTTTGGTATACAACAGCACTTAATTTAATTTAATTTGAATGTAAACATTTCTTTAAAATATTCAGTTAATGACTGTTGATGCCAAAGTCTTTTCTTTTTGCTCTTTCCAGAC
AGGGTTTCTTTCTTTGTTTAGCCTCAACTGTCCAGGAACTAGCTCTGTAAGACCAGGCTGACCTCAAACTCACAGATATCTGCCTGTTTGTTTCTGCCTCCTGAATACTGGAATTAAAGGCTTGCACCA

CCTCTGCCTGAGATGCCAAACTCTTATTGTTGACTTTTTGGTTGTGAGCCTATCCTTTAATGGCTGAGCCATCTCTCCAGCCCTAATGTTGACTTTTTAATTCAGGAAAGGCATAAATATATCAGTACA
TCTAAGAACCATTCTAGAAAAGTCGGTTCTTAGGTTTATCATTAAAAAAACAACCAACACACTAAAATTTTAAATTATCTCTTACAGAACTTGTATACATAAGGGCTATTCATTGTTATTTTAGCCTG
AAATATAGTAAAAAACAGGAGTGCTATTTTTCTCTGATTACAGAATGAGATAAAAATCTCAAGAAAGGTAATTTCTCTTGACTTTTGATGAGATCAGTGCTTAATTTCATTCCTAGTCCTGTCTCTTTT
AACCTTATGTAAAATATACCCATTCTTTTTTTGCTTCCCATGAAGCAACCTTCACATAACTATTACTATTATGACCTCTTTGTTTTGCTACAAGAACCCAATTTAAAATAACAAAAATTTATGGAAATT
AATACAAGTTTTAAAAAATGCTCAGCGGTTATTGATAGTTTGGTTGCTATATATCTTTGAAACTAAAGCTCACACAACCCATAATTCTTCATGTCTTTTTTCATTATTTTTTAATCTTTTTCTTTCACC
TATTATATGTAAATGGGGCATTTATTTTTCTAAGTCTGTGTTAATGCTAAGTCTCATGTCTCTTTACCATCAGAGAGTTACTTTCTTTTATGCTTTTACCACCATTGTTTACAGGTTTTCTTTGAGACT
CTTGGAAGATAAAGTAGAAGTGCAGGGCAGAACATCAGACATATCTTTGCTACTGAGCTCTAAATAGAGCAGGCTTGATAGTACTTTTCATAGCATTTGAAAATGAATTTAATTTAAAGATGAAAAGAA
AACAAATGTCTGGTTCAGTATTTGAGGGCAAAGATATAGAAAATCATGGATTGGCATGAAGTCTTGAGAACTCTTCTACTTTCTCTGTGAAAATGTATTAATGCATTGTTTAAAGATATCAGCTATAAC
TTTTGCCTGTATTAGAAATTGTGATATATATATGTATATATGTGTATATACATATAGATACACACACATATATGTATATATATATGATACATGAATGCATATATGTATACACACACTCACACACTCATT
CATAAACTTTGAAGCTGAGCCAGAGTGAATTATGCTTCATTTATGTGTATTACATTTCTTTTGAAACTTACAATTTTCCTGATAATGTATACTTTTTTAAGGTATATAAGTAGAGGTTTTCAGCATTCT
AAATTTGGAAGAACTTTTAAAGAAATAAAATTTGTTTAAATGTTTCTTGTTTTGTAATATCTAGACTTGTAAATAAATGATGGATTTGTTGGTAGAGTGGATATAATTTGAATGTAATAAAAATACTGT
TGAATAAATGAATTTTAAATATACTTATCAAATGAAATA

>10xBulge

GAGACAGGGTTTAACTCTTGTAGCCTGAGCTGTTCTAGAACTCACTGGGTAGACCGGGCTGGCATCAAACGTGGCCTCTCAAGTTCTGGGATTAAAGGCGTTTGTCACCACCGCCTCCCCAACTCATGA
GAGGTTCCTCTTTATACTTAATCTTGCCACAAAACAGAACTATTCTCATCGCTATGCGTTCCACATCCACGGTGGGCTCTTCCCTAAAACTGTGAGCACTTCAGTAACTATCATGTAAGCATTTCATTT
TGGGGACGATCATTACTCTTTTGGGTTGCGAGGTAAAAGAGTTAAATCTTTATTTCGGGTAATCGAGTTTTCAATGTGTCTCAGAATGAAAGATATATACTAAAAGGTTATGCGTTATTTAAAATCAAA
CCAGTCGGTCCCCTCACTGTACTTACTTTGCTCCTACTAGACCACGAACCCCAGAATTGCGGGGAAAACCCTTGTTTCCAGCGCGTTCGAGTTTGTGACTCTAGCGCTGGGCAGAAGAAGCCCGTTATA
AAGAAAGGAGCGAAGCATTAGATGATCGAGTGGGTAACCGAAATAATATGTCCTCCAATTAAATGACACTTTTGGAGAACCAAAGCCTCGGGGTCGTGAGGACCACAGCACCCCTCCAAGGACCGCATT
CCCCAGCGCAGCACCCGCACAGCCAGCGCTCCCGCGTTCTGGGGTCGCGCGCGGCGCGCCCTCGTGACGTCATCACGCGCGCGCCCGCCCGCCCGCCCGCAGCTTCTCTGGGTTAGAGTGGTGCCGGCG
CTTTCGCTTCCACCGCTGCCGTGAGCGGCGCTTTCCCGCCGGTCGCCCCCGGCGCTGTCTTCCGAGCGTTCAGTCCGCCGCGTCCTCACCGGCTCTCTTGTCTCTGTCACTGCTCGCTGGCAGCAGGCC
TGGCCGCTCCCACGCGGAGCGAGCTTCTCCTCAGGGCCATGTCCAGGCCGAGCAGCGTCTCGCCGCGTCCGCCGGCTCCTAGCGGAGGCGGCACGGGCGGCGGTGGAGGTGGCAGTGGCGGCGGCGGTG
GCGGTGGCGGCGGCGGCCCAGCTTCGTGTGGTCCTGGAGGCGGTGGCCGGGCCAAGGGCCTCAAAGACATTCGTATCGACGAGGAGGTGAAGATCGCCGTCAACATCGCTCTGGAGCGCTTCCGCTACG
GTGACCAGAGAGAAATGGAATTCCCTTCTTCTTTGACCAGTACGGAAAGAGCCTTTATTCACCGACTAAGTCAATCTCTTGGTTTAGTTTCTAAAAGTAAAGGAAAGGGAGCAAATAGATACCTAACTG
TGAAGAAGAAAGATGGATCAGAAACAGCTCATGCAATGATGACCTGTAATTTGACTCATAACACAAAACATGCTGTTAGGAGCCTAATTCAGAGATTTCCTGTCACCAATAAAGAACGTACTGAACTCC
TACCTAAAACAGAAAGAGGAAATGTGTTTGCAGTTGAAGCTGAAAACCGAGAAATGAGCAAGACAAGCGGGCGACTCAACAATGGCATTCCTCAAGTTCCTGTGAAAAGGGGAGAGTCTGAATTTGACT
CATTCAGGCAGTCTTTACCAGTTTTTGAGAAACAAGAAGAAATTGTTAAAATAATTAAGGAAAATAAAGTAGTTTTGATAGTAGGAGAAACTGGGTCTGGAAAGACCACACAGATTCCTCAGTTCCTGT
TAGATGATTGCTTTAAAAATGGCATCCCCTGCCGAATATTTTGTACTCAACCAAGACGACTAGCAGCGATTGCTGTGGCTGAAAGAGTTGCTGCAGAGAGAAGAGAAAGAATTGGTCAAACAATTGGTT
ATCAGATCCGATTAGAAAGCAGGGTTTCGCCAAAGACACTTCTGACATTTTGCACTAATGGGGTATTGCTTCGTACATTGATGGCAGGTGATAGCACATTGTCAACTGTGACCCATGTTATTGTGGATG
AAGTGCATGAAAGGGATCGATTCAGTGATTTTTTGCTTACTAAGTTAAGAGATTTGTTGCAAAAGCACCCAACTTTGAAACTAATTCTTTCGAGTGCTGCCTTGGATGTGAACCTCTTCATAAGATATT
TTGGAAGTTGTCCAGTGATATATATATACAGGGAAGACCATTTGAAGTAAAAGAAATGTTTCTGGAAGATATTTTAAGAACTACAGGATATACCAACAAAGAAATGTTAAAGTACAAAAAGGAAAAGCAAC
GAGAGGAGAAACAACAGACCACCCTCACAGAGTGGTACTCAGCTCAAGAGAATACCTTCAAGCCTGAGTCTCAGAGGCAGAGAGCTGTTGCTAGTGTGTCTGAAGAGTATGACTTATTGGATGATGGAG
GTGATGCTGTCTTCAGTCAGCTGACAGAAAAAGATGTGAATTGCCTTGAACCATGGTTAATCAAGGAGATGGATGCCTGCCTTTCTGACATATGGCTGCATAAAGATGTTGATGCTTTTGCTCAGGTCT
TTCATCTTATTTTAACTGAAAATGTTAGTGTTGATTATAGGCATAGTGAGACCAGTGCAACAGCTCTGATGGTTGCTGCAGGACGGGGCTTTACGAGCCAAGTAGAGCAGTTAATTAGTATGGGAGCCA
ATGTCCACAGTAAAGCATCAAATGGCTGGATGGCTTTAGATTGGGCTAAGCACTTTGGACAGACTGAAATTGTGGATCTTCTAGAATCTTACAGTGCTTCATTGGAATTTGGAAATCTAGATGAAAGTT
CCTTGGTTCAGACAAATGGGAATGACCTCAGTGCAGAAGACAGAGAGCTGTTGAAAGCTTACCATCATAGTTTTGATGATGAGAAAGTAGACTTGGATTTGATCATGCATCTCCTATATAATATCTGCC
ATAGCTGTGATGCTGGTGCAATATTAATTTTTCTACCTGGATATGATGAAATTGTTGGTTTGAGGGATCGTATCCTCTTTGATGACAAGCGGTTTGCTGACAATACACATAGGTATCAAGTCTTTATGC
TTCATTCAAATATGCAGACATCTGATCAAAAGAAGGTATTAAAAAATCCACCTGCAGGTGTCCGAAAAATAATCCTTTCCACCAATATTGCTGAGACGAGCATCACTGTCAATGATGTTGTGTTTGTTA
TTGATTCTGGTAAGGTGAAAGAGAAATCCTTTGATGCACTGAATTTTGTTACAATGTTAAAAATGGTATGGATTTCCAAAGCTAGTGCAATACAGCGCAAAGGCAGAGCGGGACGGTGTAGACCTGGAA
TTTGTTTCCGTCTGTTTAGTAGACTTCGATTTCAGAACATGCTGGAATTTCAGACGCCAGAACTCTTGAGAATGCCATTGCAGGAACTTTGTTTACATACCAAGCTGTTAGCCCCAGTTAACTGCACCA
TTGCCGACTTCCTTATGAAAGCCCCTGAACCGCCTCCAGCTTTAATTGTGAGAAATGCTGTGCAGATGCTTAAGACAATAGATGCAATGGATGCATGGGAAGATCTGACTGAACTTGGGTATCATTTGG
CTGATTTGCCAGTAGAACCGCATCTTGGGAAAATGGTCCTGTGTGCAGTGGTTTTGAAGTGTCTGGACCCCATCCTTACAATTGCCTGCACGCTCGCTTACCGTGACCCTTTTGTCCTGCCTACCCAGG
CCTCTCAGAAACGGGCAGCTATGCTTTGTAGGAAACGCTTCACTGCAGGGACTTTCAGTGACCACATGGCACTTCTGAGAGCATTTCAGGCATGGCAGAAAGCACGAAGTGATGGGTGGGAGCGAGCCT
TTTGTGAAAAGAATTTTCTTTCACAAGCTACTATGGAAATCATTATAGGCATGAGAACACAGTTGCTTGGTCAGCTTAGAGCATCAGGTTTTGTTAGAGCACGAGGTGGTGGTGACATTCGAGATGTTA
ATACAAACTCCGAGAACTGGGCTGTTGTTAAAGCTGCATTGGTAGCAGGCATGTATCCTAATTTAGTCCACGTGGACAGAGAGAATGTAATATTGACAGGGCCAAAGGAAAAGAAAGTACGCTTTCATC
CCACTTCAGTTCTCAGTCAACCTCAATACAAAAAGATTCCTCCCGCCAATGGTCAGGCTGCAGCGATTCAGGCATTGCCCACAGATTGGCTTATTTATGATGAAATGACCAGAGCCCATCGAATTGCTA
ATATCAGATGTTGTTCAGCAGTGACACCTGTTACTGTCCTGGTGTTCTGTGGACCAGCAAGACTGGCAAGTAACGCTCTTCAGGAACCTTCATCCTTTAGAGCGGATGGTATTCCCAATGACAGCAGTG
ACAGTGAGATGGAAGACAGAACTACTGCCAATTTGGCTGCTTTGAAGCTTGATGAATGGCTTAACTTCAAACTAGAGCCAGAGGCTGCCAGTTTATTGCTACAGCTCAGACAGAAGTGGCATAGTTTGT
TTTTACGCCGAATGAGAGCGCCATCTAAACCTTGGTCTCAAGTCGATGAAGCTACCATACGAGCAATTATAGCTGTTTTAAGCACTGAAGAACAGTCTGCAGGTTTACAGCAGCCATCTGGAATTGGCC
AGCGGCCAAGACCCATGTCTTCAGAAGAACTTCCTTTGGCTTCATCTTGGAGATCCAATAATAGTAGGAAAAGTACAGCTGACACTGAGTTTGCGGATGGATCTACTACTGGAGAAAGAGTACTGATGA
AATCTCCATCTCCAGCCTTACATCCACCTCAGAAGTACAAAGATAGAGGAATTTTACATCCTAAGCGGAGCACCGATGACCGGTCCGATCAATCATCTGTGAAATCCACAGACAGCAGCAGTTACCCAA
GTCCTTGTGCCAGCCCCTCTCCTCCATCCTCAGGGAAGGGATCAAAATCTCCTTCACCAAGACCAAACATGCCTATTCGTTACTTCATAATGAAGAGTAGCAATCTGAGAAACCTTGAAATTTCTCAAC
AGAAGGGTATCTGGTCTACAACCCCTAGTAATGAGCGGAAGCTAAATCGAGCGTTTTGGGAGAGCAGCATGGTTTACTTGGTATTTTCTGTTCAAGGATCTGGACATTTCCAGGGATTTTCTAGGATGT
CTTCTGAGATAGGAAGGGAGAAGAGCCAGGACTGGGGGTCAGCTGGACTAGGTGGAGTATTTAAAGTGGAGTGGATACGCAAAGAAAGCCTTCCCTTTCAGTTTGCACACCATTTACTCAATCCTTGGA
ACGACAACAAGAAAGTACAGATAAGCAGAGATGGGCAGGAGCTAGAACCTCAGGTTGGGGAACAGTTGCTCCAGTTATGGGAACGCCTTCCATTGGGAGAAAAAACAACTTCTGATTGACATTCAGAAC
TCCTGGCTATGGAAGAGCGTTATGCCTAAAGCTATCTGAGCACCTGACTTCATGTCACGCCAGCTGGCGTAAAAAGCTATCTGAGCACCTGACTTCATGTCATAGCGAAGAGGCCCGAAAGCTATCTGA
GCACCTGACTTCATGTCACACCGATCGCCCTTCAAAGCTATCTGAGCACCTGACTTCATGTCACCAACAGTTGCGCAGAAAGCTATCTGAGCACCTGACTTCATGTCACCTGAAAGGCGAAAGAAAGCT
ATCTGAGCACCTGACTTCATGTCAGCGCTTTGCCTGGTTAAAGCTATCTGAGCACCTGACTTCATGTCATCCGGCACCAGAAGCAAAGCTATCTGAGCACCTGACTTCATGTCAGGTGCCGGAAAGCTG
AAAGCTATCTGAGCACCTGACTTCATGTCAGCTGGAGTGCGATCTAAAGCTATCTGAGCACCTGACTTCATGTCAGTTGTAGCTACAGAATGCACTGATTTTCTGAAAACCTTGATGAAGTGTTGTATG
CCATGAGTGGATTTGAGGCATAGAGTATTCCTTCAGAATCATCATCTTCATATAGAGGAAAAGATAATTTAAATTTTTCTAGTGGTTTTAAGGAAATTATTATATAATGTTTGTTAATTATCTGTTTCA
GATGAACAAAATAGCAATTTTGGTATACAACAGCACTTAATTTAATTTAATTTGAATGTAAACATTTCTTTAAAATATTCAGTTAATGACTGTTGATGCCAAAGTCTTTTCTTTTTGCTCTTTCCAGAC
AGGGTTTCTTTCTTTGTTTAGCCTCAACTGTCCAGGAACTAGCTCTGTAAGACCAGGCTGACCTCAAACTCACAGATATCTGCCTGTTTGTTTCTGCCTCCTGAATACTGGAATTAAAGGCTTGCACCA
CCTCTGCCTGAGATGCCAAACTCTTATTGTTGACTTTTTGGTTGTGAGCCTATCCTTTAATGGCTGAGCCATCTCTCCAGCCCTAATGTTGACTTTTTAATTCAGGAAAGGCATAAATATATCAGTACA
TCTAAGAACCATTCTAGAAAAGTCGGTTCTTAGGTTTATCATTAAAAAAACAACCAACACACTAAAATTTTAAATTATCTCTTACAGAACTTGTATACATAAGGGCTATTCATTGTTATTTTAGCCTG
AAATATAGTAAAAAACAGGAGTGCTATTTTTCTCTGATTACAGAATGAGATAAAAATCTCAAGAAAGGTAATTTCTCTTGACTTTTGATGAGATCAGTGCTTAATTTCATTCCTAGTCCTGTCTCTTTT
AACCTTATGTAAAATATACCCATTCTTTTTTTGCTTCCCATGAAGCAACCTTCACATAACTATTACTATTATGACCTCTTTGTTTTGCTACAAGAACCCAATTTAAAATAACAAAAATTTATGGAAATT
AATACAAGTTTTAAAAAATGCTCAGCGGTTATTGATAGTTTGGTTGCTATATATCTTTGAAACTAAAGCTCACACAACCCATAATTCTTCATGTCTTTTTTCATTATTTTTTAATCTTTTTCTTTCACC
TATTATATGTAAATGGGGCATTTATTTTTCTAAGTCTGTGTTAATGCTAAGTCTCATGTCTCTTTACCATCAGAGAGTTACTTTCTTTTATGCTTTTACCACCATTGTTTACAGGTTTTCTTTGAGACT
CTTGGAAGATAAAGTAGAAGTGCAGGGCAGAACATCAGACATATCTTTGCTACTGAGCTCTAAATAGAGCAGGCTTGATAGTACTTTTCATAGCATTTGAAAATGAATTTAATTTAAAGATGAAAAGAA
AACAAATGTCTGGTTCAGTATTTGAGGGCAAAGATATAGAAAATCATGGATTGGCATGAAGTCTTGAGAACTCTTCTACTTTCTCTGTGAAAATGTATTAATGCATTGTTTAAAGATATCAGCTATAAC
TTTTGCCTGTATTAGAAATTGTGATATATATATGTATATATGTGTATATACATATAGATACACACACATATATGTATATATATATGATACATGAATGCATATATGTATACACACACTCACACACTCATT
CATAAACTTTGAAGCTGAGCCAGAGTGAATTATGCTTCATTTATGTGTATTACATTTCTTTTGAAACTTACAATTTTCCTGATAATGTATACTTTTTTAAGGTATATAAGTAGAGGTTTTCAGCATTCT
AAATTTGGAAGAACTTTTAAAGAAATAAAATTTGTTTAAATGTTTCTTGTTTTGTAATATCTAGACTTGTAAATAAATGATGGATTTGTTGGTAGAGTGGATATAATTTGAATGTAATAAAAATACTGT
TGAATAAATGAATTTTAAATATACTTATCAAATGAAATA

Inserted sequence
