# Supplementary material for: In vivo PIWI slicing in mouse testes deviates from rules established in vitro
Source: RNA. 2023 Mar;29(3):308–16. doi: 10.1261/rna.079349.122 (PMC9945443; doi:10.1261/rna.079349.122)
Supplement: Supplemental Material [file supp_079349.122_Supplemental_Figure_Legends.docx]

**SUPPLEMENTARY FIGURE LEGENDS**

**Supplementary Figure 1. Testicular gene expression of *10xPerf* and *10xBulge* lines**

1. Gene expression changes in *10xPerf* are shown in volcano plot with significantly dysregulated genes in red (padj <0.1). Z-scores of log2 expression of the dysregulated genes are shown in populations of spermatogonial stem cells (SSC), spermatocytes (SC) and spermatids (RS) as heatmap and boxplot.
2. Gene expression of selected genes in wild-type, *10xPerf* and *10xBulge* lines.
3. Gene expression changes in *10xBulge* are shown in volcano plot with significantly dysregulated genes in red (padj <0.1). Z-scores of log2 expression of the dysregulated genes are shown in populations of spermatogonial stem cells (SSC), spermatocytes (SC) and spermatids (RS) as heatmap and boxplot.

**Supplementary Figure 2. The *Ythdc2* reporter sequences.** Inserted sequences within the endogenous genomic sequences are highlighted in turquoise.
